# Alteration of the Morphological and Physicochemical Characteristics of Corn and Wheat Starch via Dry Heating with Whey Protein Isolates

**DOI:** 10.3390/foods13223701

**Published:** 2024-11-20

**Authors:** Eda Adal, Tugba Aktar, Hasene Keskin Çavdar

**Affiliations:** 1 Department of Nutrition and Dietetics, Faculty of Health Sciences, Çukurova University, Adana 01330, Turkey; 2 Department of Food Engineering, Faculty of Engineering, Alanya Alaaddin Keykubat University, Alanya 07450, Turkey; 3 Department of Food Engineering, Faculty of Engineering, Gaziantep University, Gaziantep 27310, Turkey; hasenekeskin@gantep.edu.tr

**Keywords:** dry heating, starch modification, wheat starch, corn starch, whey protein

## Abstract

This study investigated the impact of whey protein isolate (WPI) addition on the dry heat modification of corn (CS) and wheat starch (WS). Starches were treated under dry heating conditions at 130 °C for 2 and 4 h. The physicochemical and structural properties of the modified starches, such as color, particle size, thermal behavior (DSC), crystalline structure (XRD), and surface morphology (SEM), were analyzed. The results show that adding WPI significantly altered the gelatinization properties, surface morphology, and crystalline structure of both starches. DSC indicated that the gelatinization properties of starch/WPI mixtures varied, with corn starch showing a decreased gelatinization temperature and increased enthalpy, whereas wheat starch exhibited a more complex response, likely due to different structural changes. The XRD and FTIR results revealed WPI-enhanced crystallinity and structural changes, highlighting WPI-induced aggregation. Wheat starch, in particular, exhibited stronger interactions with WPI than corn starch, as evidenced by the accumulation patterns in the SEM images. The oil-binding capacity of native starches increased with dry heating and WPI addition, suggesting an improved hydrophobicity of starch granules. Dry heating and WPI addition significantly altered starch properties, highlighting the potential of thermal modulation to enhance starch–protein systems for targeted food applications.

## 1. Introduction

Starch is an ancient food component that humans and their ancestors used to consume for energy. The oldest written source of starch used as a food ingredient dates back to 23–74 AD [1]. To date, starch has been a well-consumed and well-used food source for both households and the food industry. Starch is extensively used in industrial and food applications as a gelling agent, thickening agent, bulking agent, hygroscopic agent, colloidal stabilizer, and adhesive material [2]. Nevertheless, for many applications, native starch has certain limits such as low interactions with water at temperatures below the gelatinization point, low shear stability, and a high tendency for retrogradation. To overcome these inherent limitations, natural starch is frequently modified for desired applications.

Seeds, roots, and tubers are very rich in terms of starch content. Corn, wheat, rice, and potato are the main sources of starch. Native starch is used as a raw material for modifiers, sweeteners, and ethanol (polyols, organic acids, and amino acids) [1]. However, starch in its native form is not suitable for most processes due to poor solubility, unstable structures, especially at high temperatures, high shear forces, weak paste consistency, and retrogradation possibilities [3,4,5]. Therefore, starches are modified to increase their functional properties and decrease their negative effects. Starch modifications typically include chemical, physical, and enzymatic techniques [6,7]. Chemical modifications include esterification, etherification, or oxidation of the hydroxyl groups. On the other hand, physical modifications involves pregelatinization [8,9,10], heat–moisture treatment [11,12,13], cold water swelling [14,15], and annealing with moist heat and dry heat [16,17]. Chemical modifications are blamed in studies for their risk of toxicity due to the reactive compounds that can be transferred to foods during processing [7,18,19]. Conversely, physical modification techniques provide safer options, including pregelatinization, heat–moisture treatment, and dry heat treatment. Dry heating has attracted interest as a “green” and “eco-friendly” technology because of its simplicity, safety, and absence of pollution. It efficiently changes starch characteristics without promoting granular integrity, allowing it to be a viable approach to satisfy the growing need for clean-label, nutritional, and functional food alternatives that attract health-conscious customers [20].

Dry heating treatment is defined as the physical method of modification under no (or minimal) moisture conditions [5,6]. Despite the selection of modifications, few studies suggest that the addition of proteins to the modification process has some promising contributions [21,22,23]. These studies investigated soy protein isolate additions [7,22,23], glutenin and gliadin additions to potato starch [21], and whey protein additions to rice starch [5]. A recent study performed by Zhu and colleagues (2020) revealed that starch modification alone has some inferior properties to those of nonstarch hydrocolloids such as protein isolates [5]. Therefore, the goal of the present study was to determine the morphological and structural effects of starch modification with whey protein isolate via dry heat treatment. To our knowledge, whey protein isolate has not been tested for its effect on corn and wheat starch modifications. Whey protein is the soluble protein fraction of dairy milk [24]. Whey protein isolate (WPI) is a whey protein product that contains 90% protein [25]. Whey proteins exhibit higher solubility among protein isolates, resulting in broad acceptability for various applications. In recent years, an increased focus on exogenous protein fortification, lowering the glycemic index, and the functional modification of starchy meals has increased the significance of studies on the combination of starch and whey protein. These studies have focused mostly on the interactions between starch and whey proteins in simple blends and their impact on the rheological, pasting, thermal, gelling, microstructural, and digestive characteristics of the blend system [5,26].

Corn and wheat starches are widely utilized in the food industry because of their availability and low cost. The importance of corn and wheat starches extends beyond their functional enhancements; they play a vital role in a wide range of formulations and various food applications, from sauces and dressings to snacks and desserts. However, they often require modification to enhance their functional properties, such as thermal stability, water solubility, and resistance to retrogradation. These modifications are essential as they improve the performance of starches in various applications, including gluten-free products and baked goods, where textural and structural integrity is crucial for consumer acceptance. The ratios of amylose to amylopectin and the granule shapes of wheat and maize starches vary, affecting their function and behavior during processing. Corn starch, distinguished by its high amylose content, is commonly employed to increase gel strength and retrogradation. Conversely, wheat starch, with a more balanced amylose–amylopectin ratio, yields softer textures and unique gelling characteristics [27,28,29].

Considering that the effects of dry heating on starch modification with protein have rarely been reported, in the present study, dry heating was carried out on corn and wheat starch with whey protein isolate for 2 and 4 h at 130 °C to systematically elucidate the impacts on the thermal properties, distinctive characteristics, microstructure, and subsequent correlation with the functionality of starch. The findings from this study provide a theoretical basis for the dry heat modification of corn and wheat starch, effectively addressing gaps in our understanding of starch modification via whey protein isolate and promoting its application in the food industry.

## 2. Materials and Methods

### 2.1. Materials

Corn starch and wheat starch (Dr. Oetker) were purchased from a local grocery shop. Whey protein isolate (>90%) was kindly provided by Ingredia, S.A., Arras, France. All other chemicals were purchased from Sigma-Aldrich Chemicals Co. (St. Louis, MO, USA).

### 2.2. Methods

#### 2.2.1. Sample Preparation

Starches were modified with dry heat treatment with WPI according to the method described by Qui et al. [7]. In accordance with that method, 3.00 g (dry basis) WPI was dissolved in 170.00 g distilled water for 2 h. Then, each starch sample was weighed to 97.00 g (dry basis) and mixed with WPI solution and stirred continuously at 25 °C for another 2 h. The mixed starch suspension was then dried at 40 °C in an oven until the moisture content fell below 10%. The dried starch was ground and passed through a sieve (100 mesh size). The CS (or WS)/WPI mixture was then subjected to heating at 130 °C for durations of 0, 2, and 4 h to produce the samples. The samples and codes are presented in Table 1. The native CS and WS were heated at 130 °C for 4 h to produce control samples CS-4h and WS-4h. After dry heating, the powders were immediately transferred to plastic cups and stored in a desiccator at room temperature for further analysis.

#### 2.2.2. Color

The color values of the powders were measured using the HunterLab Colorflex (A-60-1010-615 Model Colorimeter, HunterLab, Reston, VA, USA). The color values were represented using the L* parameter for darkness/whiteness, the a* parameter for greenness/redness, and the b* parameter for blueness/yellowness. The experiment was replicated three times, and the mean values were documented. The equipment was calibrated with standard reference white tiles with specific color coordinates (L* = 93.41, a* = −1.12, b* = 1.07) [30]. The equation below was used to calculate the total color difference (Δ*E*) as described by Pramodrao and Riar [31].

(1) $${\Delta E}^{*}={{{((\Delta L}^{*})}^{2}+{{(\Delta a}^{*})}^{2}+{{(\Delta b}^{*})}^{2})}^{0.5}$$

#### 2.2.3. Particle Size Distribution

Particle size is an important criterion for modified starches. The size of the particles was analyzed according to previous research [32] via a light scattering-type particle size analyzer (Mastersizer 3000, Malvern Instruments, Co., Ltd., Malvern, Worcestershire, England) with a wet dispersion unit (Hydro 3000MU, Malvern Instruments, Co., Ltd.). The starch was dispersed in distilled water and then transferred into the dispersion circulator tank (1000 mL beaker), which contained 800 mL of distilled water. The obscuration level was approximately 5.8% and the mixture was stirred at a 2500 rpm rate. The particle size was measured as follows: D (v, 0.1) represents the proportion of the sample mass consisting of particles less than that particular size, with 10% of the sample falling below this number. Similarly, D (v, 0.9) shows that 90% of the sample is composed of particles smaller than that size, whereas D (v, 0.5) signifies that half of the sample has a size smaller than that value. Mastersizer 3000 software (Malvern Instruments, Co., Ltd., Malvern, Worcestershire, England) was used for D (v, 0.1), D (v, 0.5), D (v, 0.9), and D (4,3) (volume mean diameter) calculations. The span value was calculated by the following formula:

(2) $$Span=\frac{D(v,0.9)-D(v,0.1)}{D(v,0.5)}$$

#### 2.2.4. Differential Scanning Calorimetry (DSC)

DSC was used to determine the thermal properties of the test samples. In the present study, the thermal properties were tested according to a previous publication by Ji and her colleagues [33]. Specifically, a 30% (*w*/*w*, dry basis) suspension of starch was prepared with distilled water and weighed into an aluminum DSC pan. After hermetic sealing of the instrument, the samples were kept at 4 °C for 24 h for sufficient hydration and then heated from 30 °C to 120 °C with a 10 °C increase per minute. Instrument software was used to measure the enthalpy change (ΔH) and onset, peak, and conclusion temperatures (T_O_, T_P_, and T_C_, respectively).

#### 2.2.5. Fourier Transform Infrared Spectroscopy (FTIR)

FTIR analysis was used for the assessment of chemical fingerprints in the infrared region of the electromagnetic radiation spectrum. For the present study, starch fingerprints were analyzed with FTIR according to the method previously suggested by Lu and colleagues [34]. For the analysis, the dried whey starch suspensions were mixed with KBr and ground into a fine powder, which was then pressed into slices for analysis. FTIR spectra were obtained with an FTIR spectrometer (IS50, Thermo Nicolet Corp., Madison, WI, USA) in the 400–4000 cm^−1^ wavenumber range. The obtained wavelengths were corrected and deconvolved by the software.

#### 2.2.6. X-Ray Diffraction (XRD)

XRD analysis was performed according to the method suggested by a previous research group [35]. Accordingly, starch samples were stored at 100% relative humidity for 24 h to ensure moisture equilibrium, which was then assessed using a diffractometer (Rigaku Corp., Tokyo, Japan) at 40 kV and 80 mA. XRD patterns were recorded in a 2θ scanning range of 4–40°, which were analyzed via MDI-Jade 6.0 software (Material Data Inc., Livermore, CA, USA).

#### 2.2.7. Polarized Light Microscope

A polarized light microscope was used for morphological property assessment. For this analysis, a 10% starch–water mixture at 20× magnification was observed via a polarizing microscope (Model BX51; Olympus Corp., Tokyo, Japan) with a 100 W halogen light source. A Pixera camera (Model PVC 100C, Los Gatos, CA, USA) was used to acquire the images.

#### 2.2.8. Scanning Electron Microscopy (SEM)

The surface properties of the dry-heated starch samples were observed by scanning electron microscopy according to previous research by Zhu et al. [5]. According to this method, samples were placed on a specimen holder with a broad surface and coated with gold (Au) under vacuum. The coated samples were then placed in SEM (Thermo Fisher Scientific Apreo S, Waltham, MA, USA) for the morphology images to be taken at a certain magnification.

#### 2.2.9. Oil-Binding Ability

The evaluation of the oil-binding characteristics was conducted, as stated by Zhu et al. [5]. A 0.5 g (dry basis) starch sample and 1.0 mL of medium-chain triglycerides were mixed for 30 s and stored for 1 h with 15 min mixing intervals. After 1 h, 5.0 mL of water was added and the mixture was vortexed for 1 min. This mixture was kept at room temperature until sedimentation occurred. The stable sedimentation volume (sunken oil-binding starch) was then measured as the oil-binding ability.

#### 2.2.10. Statistical Analysis

The experiments were conducted in triplicate and the results were expressed as mean value ± standard deviation. SPSS (22.0 Software Inc., Chicago, IL, USA) was used to perform a one-way analysis of variance (ANOVA). Duncan’s multiple range test was employed to assess the differences among the various samples. The significance level was selected as 95% (*p* < 0.05) to test the differences between the data.

## 3. Results and Discussions

### 3.1. Color Properties

Color is one of the most important factors that is likely to be altered with processing techniques. Moreover, starches are used for baking purposes, and starch quality as well as color affect the final visual score of the final product. In the present study, we aimed to observe the effects of the processing technique, protein isolate addition, and origin on starch quality.

Table 2 presents the L*, a*, and b* values of the samples. In terms of the measured color properties, the type of starch, duration of heating and dry heating, and WPI addition significantly affected the color of the samples. These analyses revealed that the samples with wheat starch and WPI exhibited lower L* values and higher b* values against the sample containing corn starch and WPI, which suggests a lower brightness and more yellowish characteristics. Additionally, the increasing duration of dry heat treatment resulted in lower L* values and higher b* values, which is a well-expected phenomenon with dry foods showing darker colors, mostly due to the evaporation of water from the structure [36].

The color difference (Δ*E*) revealed few changes in corn starch alone after 4 h of heating (0.49), indicating that Maillard reactions were restricted. Nonetheless, the incorporation of WPI increased the color difference to 0.65 without heating and further increased it to 1.18 and 1.87 after 2 and 4 h, respectively, indicating substantial interaction and reaction advancement. Conversely, wheat starch demonstrated a more significant color variation of 2.47 after 4 h of heating, indicating a greater vulnerability to browning. The WS/WPI combinations presented a color difference of 1.07 at ambient temperature, which increased to 4.22 and reached a maximum of 6.18 after 2 and 4 h, respectively, underscoring the synergistic effects of protein–starch interactions when subjected to heat [31,37].

### 3.2. Thermal Properties

The thermal properties were tested with differential scanning calorimetry. The gelatinization onset (To), peak (Tp), and endset (Tc) temperatures and gelatinization enthalpy (ΔH) of the native starch and starch/WPI mixture samples are expressed in Table 3. Overall, the thermal properties varied significantly depending on the type of starch and the duration of dry heating. The gelatinization onset temperatures of native corn and wheat starch were 65.45 °C and 58.40 °C, respectively. The corn starch-containing samples presented the highest gelatinization temperature and enthalpy, whereas the samples that contained wheat starch presented the lowest values. This fact about corn starch and relatively high gelatinization temperature was previously reported by Singh et al. [38]. Moreover, WPI addition to these starches caused a significant increase in the gelatinization onset temperature while decreasing the enthalpy of gelatinization for all samples at 0 h. This phenomenon may be due to the increase in molecular weight caused by WPI addition [39,40]. Furthermore, the smaller enthalpy values highlight that the duration of complete gelatinization is shorter than that of native starches. Therefore, WPI addition leads to a shorter gelatinization process.

The effect of the dry heating process on the thermal properties of native corn starch was obtained by comparing the CS, CS-2h, and CS-4h samples. According to that comparison, gelatinization temperatures (onset, peak, and endset) decreased significantly. Moreover, the enthalpy of gelatinization increased significantly with increasing dry heating duration.

According to the thermal properties, the findings of this study suggest that the duration of dry heating has an impact on the gelatinization temperature and enthalpy of starch/WPI mixtures. One can say that longer durations result in decreased gelatinization temperatures and increased gelatinization enthalpy with corn starch. Surprisingly, the opposite trend was observed for the wheat starch-containing samples, which may indicate that dry heating could affect the thermal properties of starch in a complex manner due to changes in the molecular structure and organization of the starch molecules over time. In the literature, prolonged cooking and heating processes resulted in a decrease in the gelatinization temperature and an increase in the enthalpy [41]. Furthermore, wheat starch was found to be more sensitive to heat treatment durations in terms of gelatinization behavior compared to the other starch types [42].

### 3.3. Particle Size

The results of the particle size analysis were tabulated in Table 4. These findings indicate that the particle size distribution significantly varied with the type of starch and duration of dry heating. Between the types, the corn starch samples presented a larger particle size compared to the other samples containing wheat starch. These findings were previously reported by Sun et al. [43,44], who suggested that corn starch particles are larger and irregularly shaped than other starch types. The addition of WPI, on the other hand, decreased the particle size of the samples. Furthermore, the duration of dry heating resulted in larger particle sizes than shorter heating times. With respect to the duration and effect of dry heating, recent studies have shown that thermal treatment and mechanical processes lead to variations in the particle size of starch [45].

These findings suggest that the type of starch and duration of dry heating significantly affect the particle size distribution of starch-containing products. Moreover, WPI addition can also affect the particle size to some extent. Therefore, we can emphasize that the type, process, and WPI addition are significant determinants for most food industries, especially for product development.

### 3.4. Fourier Transform Infrared Spectroscopy

**Figure 1:**
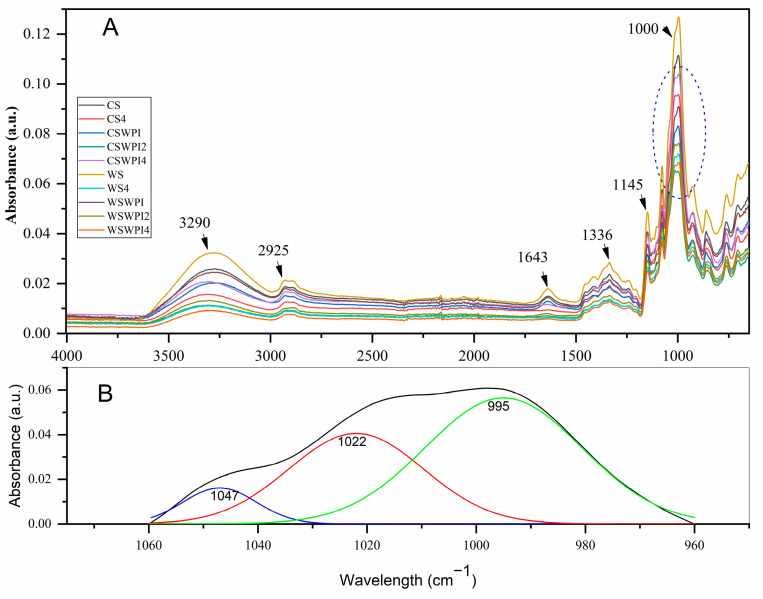
(**A**) FTIR spectra of samples of native starch and starch/WPI mixtures before and after dry heating. (**B**) An illustration of numerical deconvolution of the FTIR spectra in the starch I region (black line).

The FTIR spectra of the samples are presented in Figure 1. FTIR spectra provide information about the dry heating process and changes in the structure at a molecular level. Our findings revealed a strong absorption band for the samples, specifically at 3290 cm^−1^. This range of 3500–3400 cm^−1^ represents the O-H stretching of the starch. Furthermore, the width of the peak was attributed to the extent of intermolecular and intramolecular hydrogen bond formation [44]. The frequency range of the modified starch samples was lower than that of the native starch samples, with the band peak shifting from 3270 cm^−1^ for CS and CS/WPI-0h to 3308 cm^−1^, 3307 cm^−1^, and 3311 cm^−1^ for CS-4 h, CS/WPI-2 h, and CS/WPI-4 h, respectively. The band peak shifted from 3286 cm^−1^ for WS and WS/WPI-0 h to 3308 cm^−1^, 3307 cm^−1^, and 3311 cm^−1^ for WS-4 h, WS/WPI-2 h, and WS/WPI-4 h, respectively. These observations suggest that dry heat treatment may disrupt the hydrogen bonds between the hydroxyl groups in starch, hence increasing the quantity of nonbonded hydroxyl groups [5,33,46]. The second band that appeared was observed at 2925 cm^−1^, which is attributed to the presence of hydrocarbon chromophores [47]. A similar ordering pattern was visible for that range, with the highest for native wheat starch and the lowest for the WSWPI4 sample. The tight water bonding starch effect is attributed to showing a band at 1643 cm^−1^, which was also highest for native wheat starch and lowest for the WS/WPI-4 h sample [48].

Figure 1B illustrates the deconvolution results for the starch region, demonstrating that a precise fitting was achieved with peaks at 1047, 1022, and 995 cm^−1^. The absorption bands at 995, 1022, and 1047 cm^−1^ are characteristic of starch and represent the initial crystalline structure, the amorphous structure, and the newly formed crystalline structure after starch modification. The intensity ratio of the bands at 1047–1022 cm^−1^ serves as an indicator of the short-range order structure, reflecting both the double-helical organization and the amylose content [49,50]. The 995/1022 and 1047/1022 FTIR ratios for the various flour samples are presented in Table 5. The results indicate that the incorporation of WPI and the dry heating process adversely affect the short-range order of wheat and corn starch, with this effect being more prominent with extended dry heating durations and the subsequent addition of WPI. This can be attributed to the previously noted fact that the hydrogen bond was disrupted during the treatment process. Zhu et al. [5] reported a comparable phenomenon during the dry heat treatment of rice starch, observing that a shorter treatment duration led to a reduction in the 1047/1022 cm^−1^ ratio, attributed to the disruption of hydrogen bonds within the starch. The increase in protein content markedly elevated the 1047/1022 cm^−1^ ratio, indicating an increase in the degree of ordering of the starch molecules [51].

The addition of WPI resulted in a decrease in the 995/1022 ratio for both the CS and WS samples, which indicates a loss of water molecules. Conversely, the 995/1022 ratio exhibited a significant increase during long-term dry heating, suggesting that the hydrated structures in starch granules were enhanced for CS. The partial gelatinization of starch granules and the increased mobility of water molecules were the results of the increase in temperature. The 995 FTIR band, which is sensitive to increased hydrated structures, is a reflection of this effect [46].

### 3.5. X-Ray Diffraction

**Figure 2:**
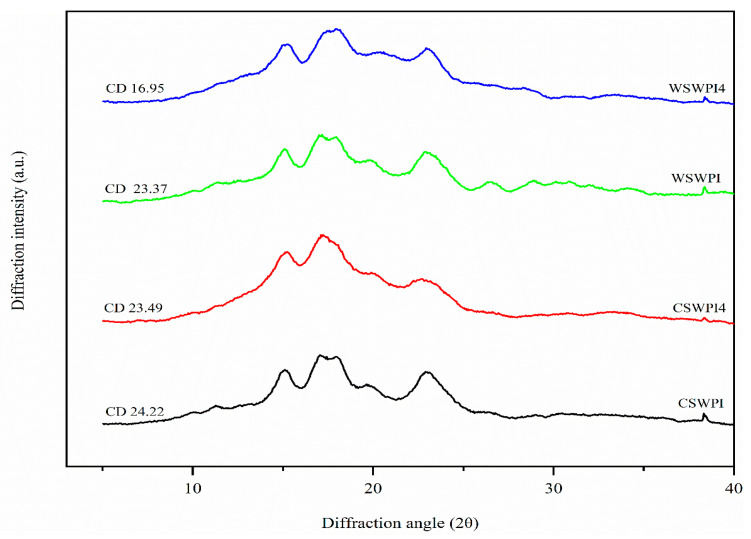
XRD patterns of CS/WPI mixture without dry heating (CS/WPI), CS/WPI mixture dry heated for 4 h (CS/WPI-4h), WS/WPI mixture without dry heating (WS/WPI), and WS/WPI mixture dry heated for 4 h (WS/WPI-4h).

The XRD patterns of the CS/WPI mixture without dry heating, the CS/WPI mixture dry heated for 4 h, the WS/WPI mixture without dry heating, and the WS/WPI mixture dry heated for 4 h are presented in Figure 2. This analysis aimed to observe WPI addition on dry heating. For the WS/WPI and CS/WPI samples that were not subjected to the dry heating process, XRD measurements showed a typical A-type crystallinity, with strong reflections at 2θ values of 15°, 17°, 18°, and 23°. In dry-heated samples, WS/WPI-4h and CS/WPI-4h, the same reflections at 2θ of 15° and 17° were observed; however, the 18° peak disappeared, while 23° decreased compared to non-dry-heated samples. A-type crystallinity did not change but decreased with the dry heating process, which is expected to change according to the gelatinization temperature and degradation of the crystalline region while increasing the amorphous content [5,7,44]. Even though we have not tested the XRD patterns of samples without WPI, WPI addition is also expected to increase the crystallinity and amorphous content, which was also observable with the SEM measurements in this study. Previous findings agree with that statement, highlighting that WPI addition increases crystallinity and has a protective effect on the starch structure [5,52].

### 3.6. Polarized Light Microscopy

**Figure 3:**
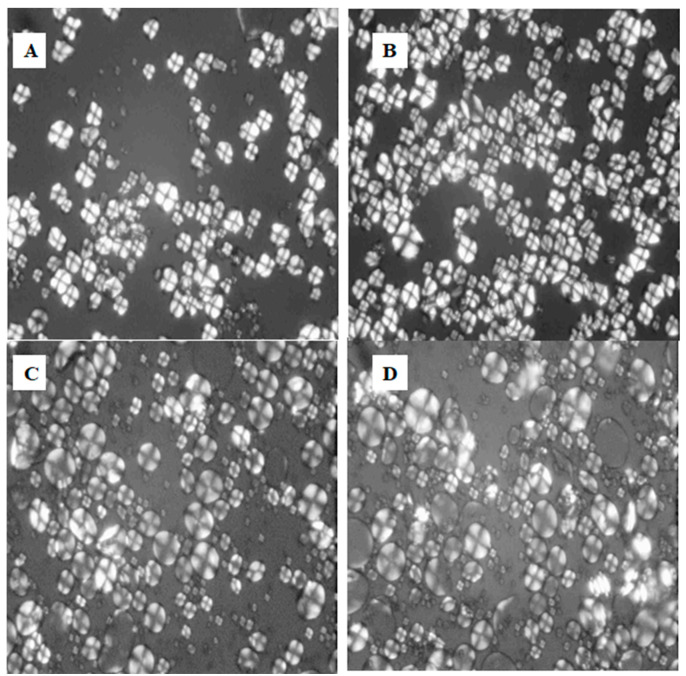
Polarized light micrographs of samples. (**A**) CS/WPI mixture without dry heating (CS/WPI), (**B**) CS/WPI mixture dry heated for 4 h (CS/WPI-4h), (**C**) WS/WPI mixture without dry heating (WS/WPI), (**D**) WS/WPI mixture dry heated for 4 h (WS/WPI-4h).

A polarized light microscope was used for structural analysis. The images are presented in Figure 3 for (A) the CS/WPI mixture without dry heating, (B) the CS/WPI mixture dry heated for 4 h, (C) the WS/WPI mixture without dry heating, and (D) the WS/WPI mixture dry heated for 4 h. In these images, the starch content showed a Maltese cross-effect due to the crystalline regions. Compared with the corn starch/WPI mixture, the wheat starch/WPI mixture resulted in larger granules. Research findings emphasize that starch type alters the granule size under a polarized light microscope by highlighting the wheat starch as larger [53,54]. Maltese crosses are visible after dry heating operation for both wheat and corn starch mixtures of WPI. The dry-heated samples (B and D) showed accumulated Maltese crosses, which means that dry heating might have an increasing effect on the amorphous regions of starch [55].

### 3.7. Morphological Properties

**Figure 4:**
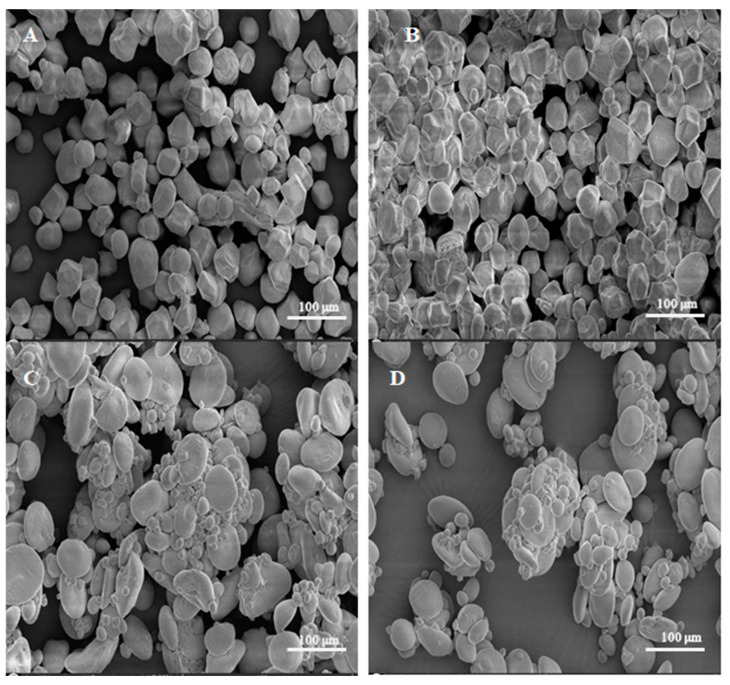
SEM micrographs of samples. (**A**) CS/WPI mixture without dry heating (CS/WPI), (**B**) CS/WPI mixture dry heated for 4 h (CS/WPI4h), (**C**) WS/WPI mixture without dry heating (WS/WPI), (**D**) WS/WPI mixture dry heated for 4 h (WS/WPI4 h).

Scanning electron micrographs are presented in Figure 4 for samples of the (A) CS/WPI mixture without dry heating, (B) CS/WPI mixture dry heated for 4 h, (C) WS/WPI mixture without dry heating, and the (D) WS/WPI mixture dry heated for 4 h. This analysis aimed to observe WPI addition during dry heating. According to the SEM micrographs, starch granules, which are expected to have smooth surfaces and clear edges [5], significantly differ when combined with WPI (Figure 4). We can also observe from the literature that WPI powder shows irregular rough bulks in its native structure [5]. For our SEM analysis, the main goal was to observe the effects of dry heating on corn starch (A) and wheat starch (C) in the mixtures not subjected to dry heat corn starch (B) and wheat starch (D) WPI mixtures for 4 h. According to these measurements, the starch–WPI combination induced an irregular surface structure which showed aggregation of the granules with the dry heating (Figure 4B–D). Aggregation was observed with the dry heating and the enlarged surfaces might have resulted from adhesion and multilayer accumulation of the WPI during the heating process [5,56,57].

To some extent, it is expected that WPI diffuses into starch granules either as surface multilayers or into starch granules via surface holes and channels. In addition to the mechanism, the literature presents important findings about the surface holes, yet interior channel diffusion still needs confirmation [58]. Moreover, the nature of our study highlights some important morphological differences between the corn and wheat starch samples. As shown in Figure 4A,C, one can highlight that the corn starch WPI mixture has less accumulation tendency compared to the wheat starch WPI mixture. The duration, therefore, has shown variation in terms of protein accumulation following dry heating. Dry heating contributes to the functionality of food proteins [59]. The present study suggests that an increasing accumulation of WPI-added dry-heated samples may have occurred during the heating process, thereby contributing to the functionality of proteins [60,61].

### 3.8. Oil-Binding Ability

**Figure 5:**
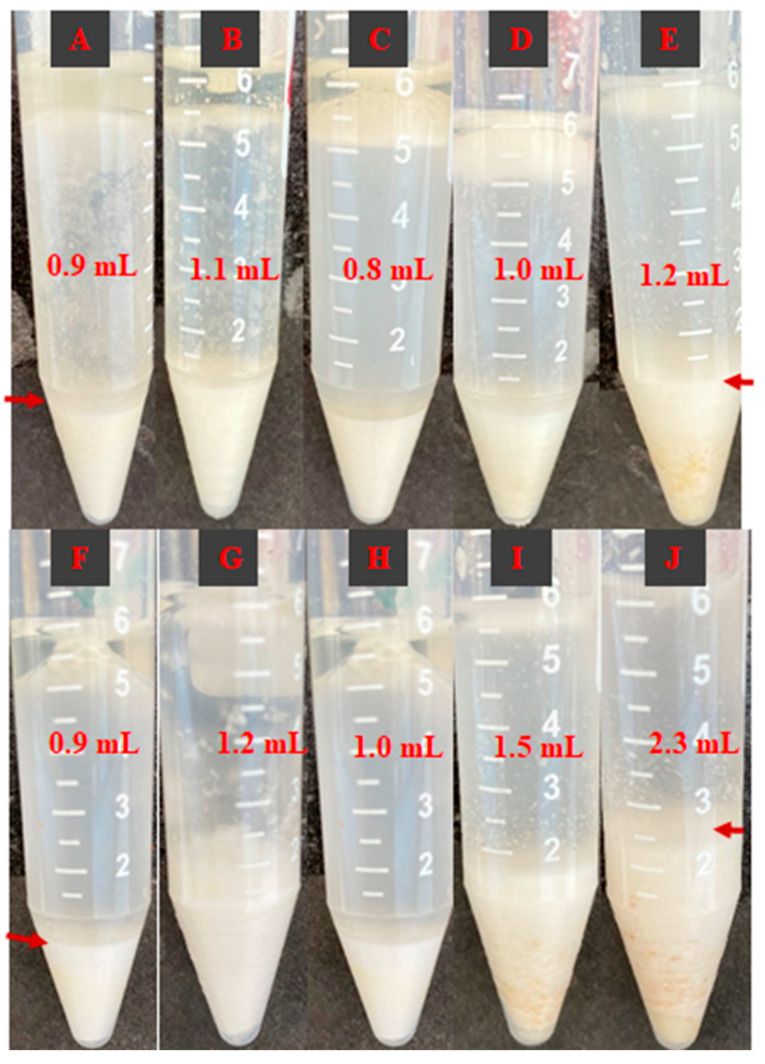
Photographs of oil-binding ability of samples. Arrow shows the interface between water and precipitate of oil–starch sample complex. Sample code: (**A**) native corn starch (CS), (**B**) corn starch dry heated for 4 h (CS4), (**C**) CS/WPI mixture without dry heating (CS/WPI), (**D**) CS/WPI mixture dry heated for 2 h (CS/WPI2h), (**E**) CS/WPI mixture dry heated for 4 h (CS/WPI4h), (**F**) native wheat starch (WS), (**G**) wheat starch dry heated for 4 h (WS4), (**H**) WS/WPI mixture without dry heating (WS/WPI), (**I**) WS/WPI mixture dry heated for 2 h (WS/WPI2h), (**J**) WS/WPI mixture dry heated for 4 h (WS/WPI4h).

Oil binding is an important measure for starch-containing foods, and it is measured by the amount of sunken oil-binding starch, as shown in Figure 5. In these photographs of the oil-binding ability of the samples, the variation in sedimentation volume at the tube was attributed to the differences in the oil–starch coagulum due to the oil-binding ability of the samples.

According to our findings, the native corn and wheat starch samples (A and F) had a 0.9 mL sedimentation volume. Similarly, after 4 h of dry heat, treated samples of corn and wheat starch (B and G) had very similar sedimentation amounts of 1.1 and 1.2 mL, respectively. WPI addition had a significant variation in the oil-binding capacities of the samples. In samples C and H, WPI added to corn and wheat starch with no dry heating process had 0.8 and 1.0 mL of sediment, respectively. The effect of dry heating duration was evident in the analysis, as longer heating times led to increased sedimentation volumes. This trend was clearly observed in the corn starch samples labeled C, D, and E, as well as in the wheat starch samples labeled H, I, and J. The literature findings agree that dry heat treatment increases the oil-binding ability of starches [5,31,62,63]. A similar phenomenon is effective with WPI addition, which increases the oil-binding ability [5].

Oil binding increases with dry heating and is even greater when WPIs are combined. This can be explained by the induced hydrophobic characteristic feature due to the dry heating technique for the endogenous and exogenous proteins in the starch structure [5]. Furthermore, WPI seems to contribute to hydrophobicity, resulting in an increased oil-binding ability. The noted increase in oil-binding ability may be advantageous for moisture-retentive applications such as sauces, baked goods, or emulsified items; however, it may be less favorable for deep-fried dishes, where excessive oil absorption poses a problem [12].

## 4. Conclusions

In the present study, the authors aimed to explore the effects of a physical starch modification method (dry heating) and process duration (2 h and 4 h at 130 °C) on two different starch types (wheat and corn) in terms of their physicochemical properties (color, oil-binding ability, DSC, FTIR, and particle size distribution) and structural characteristics (SEM, polarized light microscopy, and XRD) with formulations including whey protein isolate. The measured physicochemical and structural properties were significantly affected by the physical starch modification method, duration, and WPI addition. These findings emphasize the impact of starch type, physical starch modification method, duration of modification, and formulation of the system when thermal modulation is applied to starch-based products. This study’s findings, through additional investigations of protein–starch interactions, offer potential for extensive applications in the food and pharmaceutical sectors. Subsequent studies should concentrate on food applications to assess the performance of these modified starch–protein systems inside actual food matrices, including factors such as texture, stability, and sensory attributes. Moreover, additional research is needed to examine the impact of dry heat treatment on the molecular structure of starches and proteins, enhancing the understanding of how these alterations affect functionality and expanding their practical applications. 

## Figures and Tables

**Table 1:** Sample coding system for research on dry heating duration, starch type, and whey protein isolate addition.

Sample Code	Dry Heating Duration (h)	Dry Heating Temperature (°C)	Corn Starch Concentration (%)	Wheat Starch Concentration (%)	Whey Protein Isolate
CS	-	-	100		-
CS-4h	4	130	100		-
CS/WPI-0h	-	-	100		+
CS/WPI-2h	2	130	100		+
CS/WPI-4h	4	130	100		+
WS	-	-		100	-
WS-4h	4	130		100	-
WS/WPI-0h	-	-		100	+
WS/WPI-2h	2	130		100	+
WS/WPI-4h	4	130		100	+

**Table 2:** Color values (L*, a*, b*) and Δ*E* values of the native starch and starch/WPI mixtures before and after dry heating.

Samples	Color Values			
	L*	a*	b*	Δ*E*
CS	95.37 ± 0.00 ^a^	−0.51 ± 0.02 ^a^	5.98 ± 0.08 ^a^	0
CS-4h	95.27 ± 0.28 ^ab^	−0.62 ± 0.02 ^b^	5.66 ± 0.16 ^b^	0.49 ± 0.04 ^a^
CS/WPI-0h	95.13 ± 0.15 ^b^	−0.69 ± 0.01 ^c^	5.44 ± 0.06 ^c^	0.65 ± 0.00 ^b^
CS/WPI-2h	94.43 ± 0.08 ^c^	−0.22 ± 0.02 ^d^	6.51 ± 0.06 ^d^	1.18 ± 0.01 ^c^
CS/WPI-4h	93.88 ± 0.11 ^d^	0.10 ± 0.01 ^e^	6.75 ± 0.03 ^e^	1.87 ± 0.00 ^d^
WS	95.74 ± 0.05 ^e^	−0.12 ± 0.01 ^f^	2.27 ± 0.03 ^f^	0
WS-4h	94.16 ± 0.10 ^f^	0.55 ± 0.02 ^g^	2.62 ± 0.02 ^g^	2.47 ± 0.02 ^e^
WS/WPI-0h	94.74 ± 0.02 ^g^	−0.14 ± 0.02 ^f^	2.62 ± 0.02 ^h^	1.07 ± 0.04 ^f^
WS/WPI-2h	93.47 ± 0.05 ^h^	0.21 ± 0.02 ^h^	5.8 ± 0.02 ^i^	4.22 ± 0.01 ^g^
WS/WPI-4h	92.19 ± 0.02 ^i^	0.71 ± 0.04 ^i^	7.25 ± 0.03 ^j^	6.18 ± 0.03 ^h^

Values expressed are mean ± standard error (*n* = 3). Different letters (a, b, etc.) on the numbers in the same column indicate significant differences (*p*  <  0.05).

**Table 3:** The gelatinization temperature [onset temperature (To), peak temperature (Tp), and conclusion temperature (Tc)] and gelatinization enthalpy (ΔH) of the native starch and starch/WPI mixtures before and after dry heating.

Samples	Thermal Properties			
	To (°C)	Tp (°C)	Tc (°C)	ΔH (J/g)
CS	65.45 ± 0.05 ^a^	75.06 ± 0.06 ^a^	84.48 ± 0.02 ^a^	1.62 ± 0.01 ^a^
CS-4h	64.05 ± 0.05 ^b^	72.72 ± 0.02 ^b^	83.15 ± 0.01 ^b^	1.72 ± 0.02 ^b^
CS/WPI-0h	66.23 ± 0.10 ^c^	74.66 ± 0.02 ^c^	83.03 ± 0.03 ^b^	1.14 ± 0.01 ^c^
CS/WPI-2h	62.16 ± 0.01 ^d^	72.32 ± 0.03 ^d^	83.57 ± 0.10 ^c^	1.41 ± 0.01 ^d^
CS/WPI-4h	63.01 ± 0.01 ^e^	72.82 ± 0.01 ^e^	82.05 ± 0.05 ^d^	1.56 ± 0.01 ^a^
WS	58.40 ± 0.10 ^f^	66.65 ± 0.02 ^f^	75.04 ± 0.04 ^e^	1.16 ± 0.01 ^c^
WS-4h	60.01 ± 0.00 ^g^	70.03 ± 0.03 ^g^	78.83 ± 0.00 ^f^	1.17 ± 0.01 ^c^
WS/WPI-0h	61.16 ± 0.01 ^d^	67.03 ± 0.05 ^h^	74.50 ± 0.02 ^g^	0.97 ± 0.01 ^e^
WS/WPI-2h	57.02 ± 0.02 ^h^	65.55 ± 0.01 ^i^	73.03 ± 0.05 ^h^	1.06 ± 0.06 ^f^
WS/WPI-4h	56.02 ± 0.02 ^i^	63.34 ± 0.02 ^j^	69.12 ± 0.01 ^i^	0.78 ± 0.03 ^g^

Values expressed are mean ± standard error (*n* = 3). Different letters (a, b, etc.) on the numbers in the same column indicate significant differences (*p*  <  0.05).

**Table 4:** Particle size analysis results of the native starch and starch/WPI mixtures before and after dry heating.

Samples	Particle Sizes				
	D (v, 0.1) (μm)	D (v, 0.5) (μm)	D (v, 0.9) (μm)	Span Value	D (4,3) (μm)
CS	13.06 ± 0.1 ^a^	19.68 ± 0.1 ^a^	28.95 ± 0.5 ^a^	0.81 ± 0.1 ^a^	20.52 ± 1.2 ^a^
CS-4h	13.14 ± 0.1 ^a^	19.83 ± 0.1 ^a^	29.29 ± 0.5 ^a^	0.81 ± 0.1 ^a^	20.71 ± 1.1 ^a^
CS/WPI-0h	11.79 ± 0.2 ^b^	17.61 ± 0.2 ^b^	25.41 ± 0.3 ^b^	0.77 ± 0.1 ^b^	18.24 ± 0.9 ^b^
CS/WPI-2h	13.16 ± 0.1 ^a^	20.14 ± 0.3 ^c^	32.86 ± 1.1 ^c^	0.98 ± 0.1 ^c^	34.51 ± 2.1 ^c^
CS/WPI-4h	13.58 ± 0.0 ^a^	21.70 ± 0.3 ^d^	258.16 ± 5.8 ^d^	11.27 ± 0.2 ^d^	68.10 ± 0.8 ^d^
WS	11.31 ± 0.1 ^b^	20.23 ± 0.2 ^c^	34.10 ± 0.3 ^e^	1.13 ± 0.1 ^e^	21.91 ± 0.1 ^a^
WS-4h	14.06 ± 0.0 ^c^	27.76 ± 0.1 ^e^	54.29 ± 2.6 ^f^	1.45 ± 0.1 ^f^	32.35 ± 0.3 ^e^
WS/WPI-0h	10.72 ± 0.1 ^d^	19.13 ± 0.1 ^a^	33.40 ± 0.4 ^e^	1.19 ± 0.1 ^e^	21.06 ± 0.3 ^a^
WS/WPI-2h	15.09 ± 0.2 ^e^	31.19 ± 0.2 ^f^	178.06 ± 6.3 ^g^	5.23 ± 0.3 ^g^	97.10 ± 2.4 ^f^
WS/WPI-4h	15.92 ± 0.1 ^e^	34.74 ± 0.2 ^g^	285.17 ± 6.8 ^h^	7.75 ± 0.3 ^h^	91.40 ± 1.8 ^g^

Values expressed are mean ± standard error (*n* = 3). Different letters (a, b, etc.) on the numbers in the same column indicate significant differences (*p*  <  0.05).

**Table 5:** The ratio of band intensities at 1047–1022 and 995–1022 cm^−1^ before and after dry heating.

Samples	1047/1022 Ratio	995/1022 Ratio
CS	0.398 ± 0.001 ^a^	1.393 ± 0.001 ^a^
CS-4h	0.369 ± 0.001 ^b^	1.325 ± 0.001 ^b^
CS/WPI-0h	0.402 ± 0.002 ^a^	1.339 ± 0.004 ^b^
CS/WPI-2h	0.376 ± 0.004 ^bc^	1.281 ± 0.004 ^c^
CS/WPI-4h	0.381 ± 0.000 ^c^	1.415 ± 0.001 ^d^
WS	0.452 ± 0.005 ^d^	1.530 ± 0.009 ^e^
WS-4h	0.400 ± 0.003 ^b^	1.399 ± 0.003 ^ad^
WS/WPI-0h	0.433 ± 0.002 ^e^	1.457 ± 0.001 ^f^
WS/WPI-2h	0.374 ± 0.004 ^bc^	1.338 ± 0.001 ^b^
WS/WPI-4h	0.400 ± 0.001 ^a^	1.385 ± 0.001 ^a^

Values expressed are mean ± standard error (*n* = 3). Different letters (a, b, etc.) on the numbers in the same column indicate significant differences (*p*  <  0.05).

## Data Availability

The original contributions presented in the study are included in the article; further inquiries can be directed to the corresponding authors.
